# Latent class analysis of psychological resilience in patients with brain glioma

**DOI:** 10.3389/fpubh.2026.1899386

**Published:** 2026-09-17

**Authors:** Fengping Zhang, Jie Mei, Chuanxi Wang, Ran Duan, Dong Song, Genxiao Ding

**Affiliations:** Department of Neurosurgery, Henan Provincial People’s Hospital, Zhengzhou, Henan, China

**Keywords:** anxiety and depression, brain glioma, latent class analysis, psychological resilience, quality of life, social support

## Abstract

**Objective:**

To explore the latent class characteristics of psychological resilience in patients with brain glioma, identify influencing factors, and examine differences in psychosocial indicators across latent classes.

**Methods:**

A total of 224 patients with brain glioma hospitalized in the Department of Neurosurgery of a tertiary Grade A hospital between January 2023 and December 2024 were selected using convenience sampling. Psychological resilience, anxiety and depression, quality of life, and perceived social support were assessed using the 14-item Resilience Scale (RS-14), Hospital Anxiety and Depression Scale (HADS), European Organisation for Research and Treatment of Cancer Quality of Life Questionnaire-Core 30 (EORTC QLQ-C30), and Perceived Social Support Scale (PSSS), respectively. Latent class models were constructed using Mplus 8.3, and univariate analysis and multivariate logistic regression were performed using SPSS 26.0.

**Results:**

Psychological resilience in patients with brain glioma was classified into three latent classes: a low-level profile (25.0%), a moderate-level variable profile (32.6%), and a high-level profile (42.4%). Significant differences were observed among the classes in anxiety (*p* < 0.001), depression (*p* = 0.002), symptom burden (*p* = 0.018), global health status (*p* = 0.003), and family support (*p* = 0.018). Multivariate logistic regression analysis showed that age ≥60 years (*p* = 0.019), WHO grade III–IV glioma (*p* = 0.004), disease recurrence (*p* = 0.013), higher anxiety score (*p* < 0.001), and higher depression score (*p* = 0.032) were associated with a greater likelihood of belonging to a lower psychological resilience class, whereas college education or above (*p* = 0.027), monthly household income ≥10,000 RMB (*p* = 0.016), better global health status (*p* = 0.013), and higher family support (*p* = 0.018) were associated with a greater likelihood of belonging to a higher psychological resilience class.

**Conclusion:**

Within this clinical sample, psychological resilience in patients with brain glioma exhibited distinct heterogeneous stratification, with systematic differences in psychological status and social support across latent classes. The identification of resilience subtypes may help clinicians recognize patients who require further psychosocial assessment and may inform the development of stratified psychological support strategies. However, prospective longitudinal and interventional studies are needed to determine whether subtype-based interventions can improve psychological adaptation.

## 1 Introduction

Glioma is one of the most common primary malignant tumors of the central nervous system, characterized by high aggressiveness, frequent recurrence, and poor prognosis. Although advances in surgical techniques, radiotherapy, and chemotherapy in recent years have prolonged patient survival, the functional impairments caused by the disease itself and the uncertainties associated with long-term treatment place patients under sustained psychological stress (1–3). Studies have shown that patients with brain tumors commonly experience anxiety, depression, and reduced quality of life, with incidence rates significantly higher than those in the general population (4). Psychological resilience, defined as the capacity to maintain or regain psychological well-being in the face of adversity, is considered a key factor influencing psychological adaptation and rehabilitation outcomes in cancer patients (5). Previous studies have demonstrated that individuals with higher levels of psychological resilience are better able to alleviate disease-related stress through positive cognitive restructuring and effective coping strategies, thereby exhibiting lower levels of anxiety and depression and a higher quality of life (6, 7). In contrast, individuals with lower psychological resilience are more likely to remain in persistent negative emotional states, which may further hinder the recovery process (8). Among patients with brain tumors, psychological resilience is influenced not only by individual psychological characteristics but also by multiple external factors, including disease severity, social support, and economic conditions (9). For example, studies have shown that patients with higher-grade tumors or disease recurrence exhibit significantly reduced psychological coping capacity, whereas higher educational levels and stronger social support are associated with enhanced psychological resilience (10). However, most existing studies have primarily adopted variable-centered approaches, such as correlation or regression analyses, treating psychological resilience as a single continuous variable. This approach makes it difficult to capture the underlying heterogeneity within patient populations. In recent years, person-centered latent class analysis (LCA) has been increasingly applied in psychology and oncology. This method allows for the identification of subgroups with similar psychological characteristics, thereby revealing differences in psychological adaptation pathways among patients (11). Studies have indicated that the psychological states of cancer patients may present in diverse patterns, such as “persistently low,” “fluctuating,” and “stable and positive,” with significant differences across subgroups in emotional status and quality of life (12). These findings suggest that relying solely on mean scores is insufficient to reflect patients’ true psychological status, and that categorical approaches are more effective in accurately identifying high-risk populations. In the present cross-sectional study, however, latent classes were interpreted as contemporaneous item-response profiles rather than temporal trajectories. Nevertheless, research on the latent structure of psychological resilience among patients with brain glioma remains limited. The hierarchical characteristics of resilience and its associations with psychosocial indicators have not yet been systematically explored. Therefore, this study employs latent class analysis to classify psychological resilience in patients with brain glioma and further examines differences among identified subgroups in terms of anxiety, depression, quality of life, and social support. In addition, factors associated with resilience class membership are explored to provide preliminary evidence for stratified psychosocial assessment and to inform future research on supportive care strategies for patients with brain glioma (Figure 1).

**Figure 1:**
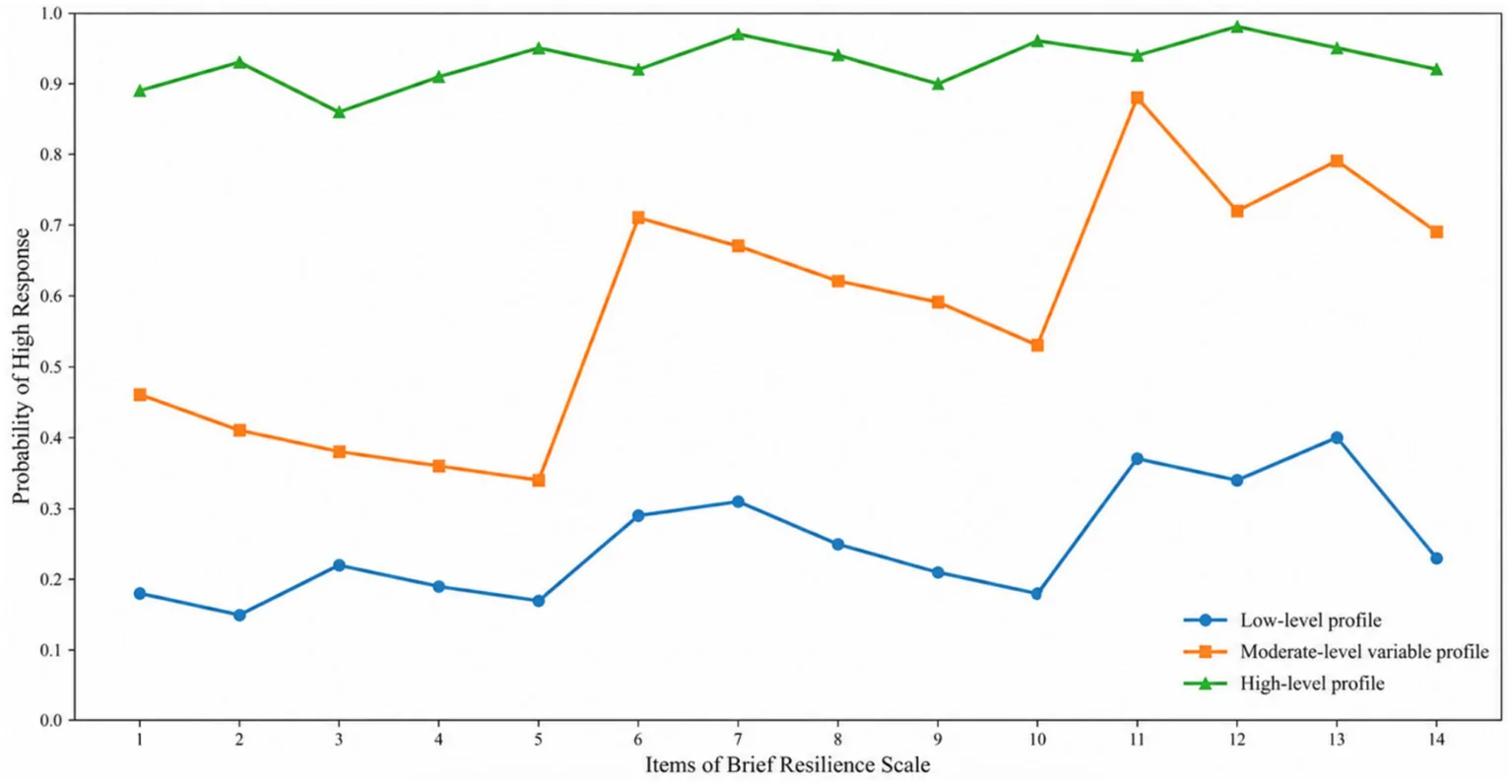
Profile distribution of latent classes of psychological resilience in patients with glioma.

## 2 Subjects and methods

### 2.1 Participants

Using convenience sampling, patients with brain glioma who were hospitalized in the Department of Neurosurgery at a tertiary Grade A hospital between January 2023 and December 2024 were recruited as study participants. During the recruitment period, patients were screened according to medical records, imaging findings, pathological results, and bedside clinical assessment of consciousness, orientation, comprehension, and communication ability. Eligibility with respect to cognitive and communication status was determined functionally according to whether the patient could understand the study and questionnaire instructions, communicate adequately with the research staff, and provide reliable responses to the self-report questionnaires. A formal neuropsychological assessment or standardized cognitive screening instrument, such as the Mini-Mental State Examination (MMSE) or Montreal Cognitive Assessment (MoCA), was not routinely administered specifically for study eligibility. Eligible patients were invited to participate when their clinical condition was stable and they were able to understand and complete the questionnaire. The diagnosis of brain glioma was based on the 5th edition of the WHO Classification of Tumors of the Central Nervous System (2021) and relevant clinical guidelines (13), and was confirmed by imaging findings, such as cranial MRI, and postoperative pathological examination results. Tumor grade was categorized as WHO grade I–II and WHO grade III–IV according to the WHO grading system. No restriction was imposed according to tumor location; patients with different tumor locations were eligible if they met the cognitive, communication, and clinical stability requirements. Ongoing or previous treatment status, including surgery, radiotherapy, and chemotherapy, was not used as an exclusion criterion but was recorded as treatment-related information. The sample size was estimated based on the requirements of latent class analysis and factor analysis. For factor analysis, according to the principles of sample size estimation for quantitative studies in nursing research (14), the sample size is generally 5–10 times the number of variables. In this study, 19 candidate variables were included; therefore, the required sample size ranged from 95 to 190 cases. Considering an estimated invalid questionnaire rate of approximately 10%, the final required sample size was 106–211 cases. A total of 240 questionnaires were distributed, of which 224 were returned, yielding a valid response rate of 93.3%. Ultimately, 224 patients were included in the analysis.

The inclusion criteria were as follows: (1) age ≥18 years; (2) confirmed diagnosis of brain glioma; (3) clear consciousness and adequate orientation, communication, and comprehension abilities to complete the self-report questionnaires; (4) clinically stable at the time of questionnaire administration; and (5) voluntary participation with written informed consent. Patients were not excluded on the basis of tumor location or ongoing treatment status alone.

The exclusion criteria were as follows: (1) cognitive impairment, disturbance of consciousness, severe aphasia, or other communication disorders of sufficient severity to prevent the patient from understanding the study or questionnaire instructions, communicating adequately with the research staff, or providing reliable responses to the self-report questionnaires; (2) current or previous severe psychiatric disorders; (3) concomitant other malignant tumors; (4) critical condition or severe postoperative complications that made questionnaire assessment inappropriate; and (5) incomplete key questionnaire data. This study was approved by the hospital’s Institutional Review Board (Approval no. 202301003). All participants provided written informed consent.

### 2.2 Methods

Data were collected using a questionnaire survey during the index hospitalization. All psychometric instruments, including the RS-14, HADS, EORTC QLQ-C30, and PSSS, were administered as patient-reported self-report questionnaires. Trained researchers distributed the questionnaires on-site in the ward and provided standardized instructions to explain the purpose, procedures, and precautions for completion. The questionnaires were completed once after eligibility confirmation, when the patients’ clinical condition was stable and they had adequate consciousness, communication ability, and comprehension ability.

For patients who underwent surgery during the current hospitalization, the questionnaire assessment was conducted after postoperative consciousness and communication had recovered and before discharge, generally on postoperative days 3–7. For patients who did not undergo surgery during the current hospitalization or were admitted for recurrence evaluation, radiotherapy, chemotherapy, or other treatment-related care, the questionnaires were completed during a clinically stable interval before the next planned major treatment session. No repeated pre- and post-treatment psychological assessments were performed in this cross-sectional study. For participants with difficulties in reading or writing, assistance was provided by the researchers without inducing responses. All questionnaires were collected immediately after completion and checked for completeness. Questionnaires with missing items were returned to the participants for timely completion.

Statistical analyses were performed using SPSS 26.0 and Mplus 8.3. Categorical variables were presented as frequencies and percentages, and comparisons between groups were conducted using the chi-square (*χ*^2^) test. Continuous variables were expressed as mean ± standard deviation (mean ± SD), and comparisons between groups were performed using one-way analysis of variance (ANOVA).

Using the 14 items of the RS-14 as observed indicators, latent class analysis (LCA) was performed using Mplus 8.3. Models with one to five latent classes were constructed sequentially and compared. Model selection was based on statistical fit indices, classification accuracy, class proportions, model parsimony, and clinical interpretability. The Akaike information criterion (AIC), Bayesian information criterion (BIC), and adjusted Bayesian information criterion (aBIC) were used to evaluate model fit, with lower values indicating better fit. Entropy was used to assess classification accuracy, with values closer to 1.00 indicating clearer class separation. The Lo–Mendell–Rubin adjusted likelihood ratio test (LMR) and bootstrap likelihood ratio test (BLRT) were used to compare the k-class model with the k − 1-class model. A statistically significant *p* value indicated that the k-class model fitted the data better than the model with one fewer class. Models with very small class proportions, poor classification accuracy, or limited clinical interpretability were not preferred. The final model was selected according to these criteria and the principle of parsimony (11).

Subsequently, the identified latent classes of psychological resilience were used as the dependent variable. Variables that showed statistically significant differences in univariate analyses and psychosocial comparisons were included in an ordinal multinomial logistic regression model to explore factors associated with latent class membership in patients with brain glioma. Because the latent classes were constructed only from the 14 RS-14 items, anxiety and depression measured by HADS were not used to define the latent classes. Instead, HADS-A and HADS-D scores were entered as external psychosocial correlates to examine whether emotional distress was independently associated with resilience class membership. Given the cross-sectional design, these variables were interpreted as associated factors rather than causal predictors. Before multivariate regression analysis, potential multicollinearity among candidate predictors was assessed using variance inflation factors (VIFs) and tolerance values. A VIF value greater than 5.0 or a tolerance value less than 0.20 was considered indicative of potential multicollinearity. The VIF and tolerance values of the predictors retained in the final model were reported in the Results section. A two-sided significance level of α = 0.05 was applied.

### 2.3 Outcome measures and instruments

#### 2.3.1 General information

A self-designed general information questionnaire and medical record review were used to collect patients’ demographic and disease-related data, including gender, age, marital status, educational level, employment status, monthly household income, method of medical payment, tumor grade, recurrence status, and treatment-related information such as surgery, radiotherapy, and chemotherapy. Marital status was recorded using the three categories available in the study dataset: single, married, and divorced/widowed. Cohabiting or other non-marital partnership status was not separately captured, and divorced and widowed participants were analyzed as a combined category. Therefore, marital status was treated as a general demographic descriptor rather than as a direct measure of the presence, quality, or availability of partner or family support. Monthly household income was categorized as <5,000 RMB, 5000–10,000 RMB, and >10,000 RMB per month according to the categories used in the study questionnaire. To provide socioeconomic context for international readers, official statistics showed that the annual per capita disposable income of Zhengzhou residents was 45,994.2 RMB in 2024, equivalent to approximately 3,833 RMB per person per month (15). This population-level per capita disposable income is provided only as a contextual benchmark and is not directly equivalent to the total monthly household income measured in the present study. Accordingly, the three income categories should be interpreted as study-specific relative socioeconomic strata rather than formal thresholds for poverty, median income, or affluence. In response to the potential influence of treatment-related and psychosocial care factors on psychological resilience, current psychological or psychiatric support and the use of commonly relevant medications during the index hospitalization, including glucocorticoids, antiepileptic drugs, antidepressants, and anxiolytics, were also reviewed from medical records and recorded as yes/no variables. Cognitive and communication status were reviewed during eligibility screening to determine whether patients could reliably understand and complete the self-report questionnaires. This screening was based on clinical information and functional bedside assessment of consciousness, orientation, comprehension, and communication ability rather than a formal neuropsychological evaluation. Tumor location was reviewed during clinical screening but was not used as an inclusion or exclusion criterion in this study.

#### 2.3.2 14-item resilience scale

The 14-item Resilience Scale (RS-14) was used as a patient-reported self-report instrument to assess patients’ levels of psychological resilience at the same questionnaire assessment time point described above. This scale was developed by Wagnild et al. based on the original Resilience Scale (RS-25) (16). The Chinese version was translated and culturally adapted by Tian Jun et al., and its reliability and validity have been validated in a Chinese population (17). The scale consists of two dimensions, namely “Personal Competence” and “Acceptance of Self and Life,” comprising a total of 14 items. Each item is rated on a 7-point Likert scale ranging from “strongly disagree” (1) to “strongly agree” (7), with total scores ranging from 14 to 98. Higher scores indicate higher levels of psychological resilience. The Cronbach’s alpha coefficient of the original scale was 0.93. For the Chinese version, the Cronbach’s alpha coefficient was 0.965 among patients and 0.961 among caregivers.

#### 2.3.3 Hospital anxiety and depression scale

The Hospital Anxiety and Depression Scale (HADS) was used as a patient-reported self-report instrument to assess patients’ symptoms of anxiety and depression at the same questionnaire assessment time point described above. This scale was developed by Zigmond and Snaith (18) in 1983. The Chinese version was translated and culturally adapted by Ye Weifei et al. (19), with a Cronbach’s alpha coefficient of 0.879. The scale consists of two subscales: Anxiety (HADS-A) and Depression (HADS-D), with 7 items in each subscale, for a total of 14 items. Each item is rated on a 4-point Likert scale (0–3 points). The score range for each subscale is 0–21, and the total score ranges from 0 to 42. Higher scores indicate more severe anxiety or depressive symptoms. Scores of 0–7 indicate no symptoms, 8–10 suggest possible symptoms, and 11–21 indicate definite symptoms. The Cronbach’s alpha coefficient of the original scale was 0.93.

#### 2.3.4 Quality of life (EORTC QLQ-C30)

The European Organisation for Research and Treatment of Cancer Quality of Life Questionnaire-Core 30 (EORTC QLQ-C30) was used to assess patients’ quality of life. This questionnaire was developed by the European Organisation for Research and Treatment of Cancer (EORTC) (20). The Chinese version was translated by Wan Chonghua et al. and has been validated for reliability and validity in Chinese cancer patients (21). The questionnaire includes multiple dimensions, such as functional status, symptom burden, and global health status. In this study, the symptom burden and global health status dimensions were selected for analysis. After linear transformation, all scores range from 0 to 100. Higher scores in functional status and global health status indicate better quality of life, whereas higher scores in symptom burden indicate more severe symptoms. The Cronbach’s alpha coefficients of the Chinese version range from 0.70 to 0.90.

#### 2.3.5 Perceived social support scale

The Perceived Social Support Scale (PSSS) was used to assess patients’ perceived levels of social support. This scale was developed by Zimet et al. (22) in 1987. The Chinese version was translated and revised by Jiang Qianjin et al. (23) and has demonstrated good reliability and validity in Chinese populations, with a Cronbach’s alpha coefficient of 0.90. The scale consists of three dimensions: family support, friend support, and other support, with a total of 12 items. Each item is rated on a 7-point Likert scale ranging from “strongly disagree” to “strongly agree” (scored 1–7), with total scores ranging from 12 to 84. Higher scores indicate higher levels of perceived social support. The Cronbach’s alpha coefficient of the original scale was 0.88.

## 3 Results

### 3.1 Univariate analysis of general characteristics and latent classes of psychological resilience in patients with brain glioma

A total of 224 patients with brain glioma were included in this study. Based on the results of latent class analysis, participants were categorized into different psychological resilience classes, and their demographic and clinical characteristics were compared. Significant differences were observed among the latent classes in age (*p* = 0.027), educational level (*p* = 0.006), monthly household income (*p* = 0.001), WHO tumor grade (*p* = 0.002), and recurrence status (*p* = 0.022). No statistically significant differences were found in gender, employment status, method of medical payment, surgical status, radiotherapy status, or chemotherapy status. Using the three-category marital-status variable available in this study, no statistically significant between-class difference was detected for marital status; the exact *p* values are presented in Table 1. In terms of distribution, the low psychological resilience group had a relatively higher proportion of older patients (≥60 years), patients with WHO grade III–IV glioma, and patients with recurrence. In contrast, the high psychological resilience group included a greater proportion of patients with higher educational levels and higher household income. Additional treatment-related and psychosocial care characteristics during the index hospitalization are summarized descriptively in Table 1.

**Table 1:** General characteristics of patients with glioma and univariate analysis of latent classes of psychological resilience.

Variable	Category	Total (*n*, %)	Class 1 (*n* = 56)	Class 2 (*n* = 73)	Class 3 (*n* = 95)	*χ*^2^/*F*	*p*
Gender	Male	117 (52.2)	27 (48.2)	39 (53.4)	51 (53.7)	0.462	0.794
	Female	107 (47.8)	29 (51.8)	34 (46.6)	44 (46.3)		
Age	≤44	54 (24.1)	10 (17.9)	17 (23.3)	27 (28.4)	10.917	0.027
	45–59	98 (43.8)	21 (37.5)	36 (49.3)	41 (43.2)		
	≥60	72 (32.1)	25 (44.6)	20 (27.4)	27 (28.4)		
Marital status	Single	21 (9.4)	6 (10.7)	7 (9.6)	8 (8.4)	5.384	0.251
	Married	187 (83.5)	43 (76.8)	63 (86.3)	81 (85.3)		
	Divorced/Widowed	16 (7.1)	7 (12.5)	3 (4.1)	6 (6.3)		
Education	≤Junior high	71 (31.7)	24 (42.9)	25 (34.2)	22 (23.2)	14.263	0.006
	High school/Technical	83 (37.1)	22 (39.3)	28 (38.4)	33 (34.7)		
	College or above	70 (31.3)	10 (17.9)	20 (27.4)	40 (42.1)		
Employment	Employed	94 (42.0)	16 (28.6)	31 (42.5)	47 (49.5)	9.126	0.058
	Retired	60 (26.8)	18 (32.1)	18 (24.7)	24 (25.3)		
	Unemployed	70 (31.3)	22 (39.3)	24 (32.9)	24 (25.3)		
Monthly household income	<5,000 RMB	76 (33.9)	28 (50.0)	27 (37.0)	21 (22.1)	18.774	0.001
	5,000–10,000 RMB	81 (36.2)	18 (32.1)	29 (39.7)	34 (35.8)		
	>10,000 RMB	67 (29.9)	10 (17.9)	17 (23.3)	40 (42.1)		
Payment	Self-paid	19 (8.5)	6 (10.7)	7 (9.6)	6 (6.3)	2.418	0.660
	Insurance	181 (80.8)	43 (76.8)	59 (80.8)	79 (83.2)		
	Other	24 (10.7)	7 (12.5)	7 (9.6)	10 (10.5)		
Tumor grade	I–II	79 (35.3)	13 (23.2)	21 (28.8)	45 (47.4)	13.855	0.002
	III–IV	145 (64.7)	43 (76.8)	52 (71.2)	50 (52.6)		
Recurrence	No	174 (77.7)	39 (69.6)	53 (72.6)	82 (86.3)	7.646	0.022
	Yes	50 (22.3)	17 (30.4)	20 (27.4)	13 (13.7)		
Surgery	No	17 (7.6)	6 (10.7)	5 (6.8)	6 (6.3)	1.520	0.468
	Yes	207 (92.4)	50 (89.3)	68 (93.2)	89 (93.7)		
Radiotherapy	No	109 (48.7)	31 (55.4)	36 (49.3)	42 (44.2)	1.676	0.433
	Yes	115 (51.3)	25 (44.6)	37 (50.7)	53 (55.8)		
Chemotherapy	No	112 (50.0)	27 (48.2)	40 (54.8)	45 (47.4)	1.042	0.594
	Yes	112 (50.0)	29 (51.8)	33 (45.2)	50 (52.6)		
Psychological or psychiatric support during the index hospitalization	Yes	18 (8.0)	—	—	—	—	—
Glucocorticoid use during the index hospitalization	Yes	96 (42.9)	—	—	—	—	—
Antiepileptic drug use during the index hospitalization	Yes	121 (54.0)	—	—	—	—	—
Antidepressant or anxiolytic use during the index hospitalization	Yes	16 (7.1)	—	—	—	—	—

Data are presented as *n* (%). Class 1 represents the low-level psychological resilience profile; Class 2 represents the moderate-level variable psychological resilience profile; and Class 3 represents the high-level psychological resilience profile. Psychological or psychiatric support and medication use during the index hospitalization are presented descriptively for the overall cohort only. Between-class comparisons were not performed for these variables because detailed information regarding treatment indication, dosage, duration, timing relative to questionnaire assessment, and adherence was not consistently available. Therefore, these variables were not included in the multivariable regression analysis. “—” indicates not analyzed. RMB, renminbi; WHO, World Health Organization. Marital status was available as single, married, and divorced/widowed; cohabiting or other non-marital partnership status was not separately recorded. The combined divorced/widowed category reflects the available data structure and should not be interpreted as indicating psychosocial equivalence between divorce and bereavement. Monthly household income refers to the total reported household income per month. The three income categories represent study-specific socioeconomic strata rather than formal thresholds for poverty, median income, or affluence. For socioeconomic context, the annual per capita disposable income of Zhengzhou residents was 45,994.2 RMB in 2024 (15).

### 3.2 Model fit of latent classes of psychological resilience in patients with brain glioma

LCA was conducted to identify latent subgroups of psychological resilience among patients with brain glioma. Models with one to five classes were fitted and compared. As shown in Table 2, the AIC, BIC, and aBIC values generally decreased as the number of classes increased, suggesting improved model fit. The three-class model showed good classification accuracy, with entropy = 0.917, and both the LMR test (*p* = 0.018) and BLRT (*p* < 0.001) were statistically significant, indicating that the three-class model fitted the data better than the two-class model. Although the four-class and five-class models showed slightly lower AIC and aBIC values, the LMR tests were not statistically significant for either model (four-class model: *p* = 0.146; five-class model: *p* = 0.092). In addition, the entropy values of the four-class and five-class models were lower than that of the three-class model, and the additional classes had relatively small proportions and did not provide clearer clinical interpretability. The three-class model yielded class proportions of 25.0, 32.6, and 42.4%, corresponding to a low-level profile, a moderate-level variable profile, and a high-level profile, respectively. Therefore, considering statistical fit, classification accuracy, class proportions, model parsimony, and clinical interpretability, the three-class solution was selected as the optimal model.

**Table 2:** Model fit indices and class proportions for latent class analysis of psychological resilience in patients with glioma.

Number of classes	Free parameters	AIC	BIC	aBIC	Entropy	LMR (P)	BLRT (P)	Class proportions
1	14	4018.764	4066.521	4023.941	—	—	—	1.000
2	30	2958.337	3057.184	2972.961	0.882	<0.001	<0.001	0.618/0.382
3	46	2816.592	2967.448	2841.664	0.917	0.018	<0.001	0.250/0.326/0.424
4	62	2810.904	3013.769	2846.425	0.861	0.146	0.039	0.103/0.247/0.228/0.422
5	78	2809.771	3064.645	2855.741	0.873	0.092	0.083	0.094/0.109/0.233/0.176/0.388

### 3.3 Distribution characteristics of latent classes of psychological resilience in patients with brain glioma

The distribution plot of item-response probabilities across latent classes showed distinct patterns among the identified groups. Overall, the response curves of each class demonstrated clear separation in both magnitude and trend, with minimal overlap, indicating good discrimination between classes. Specifically, patients in Class 1 showed consistently low response levels across all indicators, with relatively limited variation across items, reflecting a generally low level of psychological resilience. Patients in Class 2 exhibited moderate response levels across most indicators, with greater variation across items. Patients in Class 3 demonstrated generally high response levels across all indicators, with relatively limited variation across items. These findings indicate that the latent classes differ in both overall response levels and cross-item variability at the single assessment time point.

### 3.4 Naming and characteristics of latent classes of psychological resilience

Based on the results of the latent class model and the distribution patterns of item-response probabilities, the 224 patients were classified into three latent classes, with proportions of 25.0, 32.6, and 42.4%, respectively. According to the overall response levels and cross-item variation observed at the single assessment time point, the three classes were labeled as the low-level profile, the moderate-level variable profile, and the high-level profile, respectively. Specifically, the low-level profile showed consistently lower response probabilities across the RS-14 items, with relatively limited cross-item variation. The moderate-level variable profile showed intermediate response probabilities overall but greater variation across individual RS-14 items. The high-level profile showed generally higher response probabilities across the RS-14 items, with relatively limited cross-item variation. These labels describe cross-sectional item-response configurations only and do not imply stability, fluctuation, or change in psychological resilience over time.

### 3.5 Comparison of psychosocial indicators across latent classes of psychological resilience

Significant differences were observed among latent classes of psychological resilience in several psychosocial indicators, including anxiety, depression, quality of life, and social support. ANOVA results showed statistically significant differences among the three classes in anxiety (*F* = 31.842, *p* < 0.001), depression (*F* = 18.667, *p* = 0.002), symptom burden (*F* = 8.764, *p* = 0.018), global health status (*F* = 11.528, *p* = 0.003), and family support (*F* = 9.214, *p* = 0.018). No statistically significant differences were found in friend support (*p* = 0.116) or other support (*p* = 0.148). Across the three classes, anxiety and depression scores decreased progressively, whereas global health status and family support scores increased. Detailed results are presented in Table 3.

**Table 3:** Comparison of psychosocial indicators among different latent classes of psychological resilience.

Variable	*n*	Anxiety (HADS-A)	Depression (HADS-D)	Symptom burden	Global health status	Family support	Friend support	Other support
Class 1	56	10.4 ± 3.6	9.0 ± 3.1	42.8 ± 12.5	47.6 ± 13.2	20.2 ± 4.5	18.8 ± 4.7	20.4 ± 4.6
Class 2	73	8.1 ± 3.1	7.0 ± 2.9	39.6 ± 11.9	51.3 ± 12.8	22.9 ± 4.2	20.7 ± 3.9	21.5 ± 3.7
Class 3	95	5.6 ± 2.4	4.8 ± 2.1	37.9 ± 10.8	53.8 ± 11.7	25.0 ± 3.8	21.6 ± 4.2	22.0 ± 3.3
*F*		31.842	18.667	8.764	11.528	9.214	2.174	1.928
*P*		<0.001	0.002	0.018	0.003	0.018	0.116	0.148

### 3.6 Multivariate logistic regression analysis of latent classes of psychological resilience in patients with brain glioma

Using latent classes of psychological resilience as the dependent variable, demographic, clinical, and psychosocial variables with statistical significance in the univariate analysis and psychosocial comparisons were included in an ordinal multinomial logistic regression model. HADS-A and HADS-D scores were entered into the model as external psychosocial correlates of resilience class membership rather than as presumed causal predictors. Before regression analysis, multicollinearity diagnostics were performed for all variables entered into the final model. The VIF values were 1.08 for age ≥60 years, 1.43 for educational level, 1.58 for monthly household income, 1.31 for tumor grade, 1.24 for recurrence, 2.36 for anxiety score, 2.21 for depression score, 1.74 for global health status, and 1.38 for family support. The corresponding tolerance values ranged from 0.424 to 0.927. All VIF values were below 5.0 and all tolerance values were above 0.20, indicating no serious multicollinearity among the included variables. The regression results showed that age ≥60 years, WHO grade III–IV glioma, disease recurrence, higher anxiety scores, and higher depression scores were associated with a greater likelihood of belonging to a lower psychological resilience class. In contrast, college education or above, monthly household income ≥10,000 RMB, better global health status, and higher family support were associated with a greater likelihood of belonging to a higher psychological resilience class. Detailed results are presented in Table 4.

**Table 4:** Multivariate logistic regression analysis of latent classes of psychological resilience.

Variable	*β*	SE	Wald *χ*^2^	*p*	OR	95% CI
Constant 1	−1.824	0.647	8.087	0.034	—	—
Constant 2	0.957	0.591	2.595	0.107	—	—
Age ≥60 years	−0.676	0.286	5.739	0.019	0.512	0.301–0.883
Educational level (college or above)	0.784	0.317	6.323	0.027	2.181	1.191–3.982
Monthly household income ≥10,000 RMB	0.715	0.276	6.911	0.016	2.034	1.206–3.420
WHO grade III–IV glioma	−0.964	0.347	8.001	0.004	0.386	0.195–0.779
Recurrence (yes)	−0.826	0.316	6.995	0.013	0.444	0.248–0.821
Anxiety score	−0.188	0.051	13.013	<0.001	0.832	0.753–0.919
Depression score	−0.121	0.039	9.627	0.032	0.886	0.821–0.957
Global health status	0.036	0.012	9.018	0.013	1.037	1.013–1.062
Family support	0.097	0.041	5.605	0.018	1.102	1.017–1.194

Constant term 1 represents the cutoff point between the low psychological resilience group and the medium/high resilience group, while constant term 2 represents the cutoff point between the low/medium resilience group and the high resilience group.

## 4 Discussion

### 4.1 Structural characteristics of latent classes of psychological resilience in patients with brain glioma

Based on latent class analysis, this study demonstrated that psychological resilience among patients with brain glioma does not follow a single continuous distribution, but can be classified into three distinct latent classes, reflecting significant heterogeneity within the population. Considering both model fit indices and class characteristics, the three-class model showed good statistical performance and clinical interpretability, indicating the presence of a stratified structure of psychological resilience in this patient group. Comparisons of demographic and clinical characteristics revealed that patients in the low psychological resilience group were more likely to be older, have lower educational attainment and income, and present with WHO grade III–IV glioma and disease recurrence, whereas those in the high psychological resilience group were more likely to have higher educational and socioeconomic status. These findings suggest that the stratification of psychological resilience reflects not only differences in psychological status but also broader disparities in social resources and disease burden. Previous studies have described longitudinal heterogeneity in psychological adaptation among patients with brain tumors, including different trajectories of adaptation over time (24). However, such temporal trajectories cannot be inferred from the present cross-sectional data. Accordingly, the three classes identified in this study were interpreted solely according to their item-response levels and cross-item variation at a single assessment time point. Approximately one-quarter of patients belonged to the low-level profile, characterized by generally low response probabilities across the RS-14 items. The moderate-level variable profile showed intermediate overall response levels with greater variation across individual items, whereas the high-level profile showed generally high response probabilities across items. Importantly, “variable” in this context refers to variation across RS-14 items at the same assessment and does not indicate within-person fluctuation over time. These cross-sectional profiles may help clinicians recognize heterogeneity in current resilience characteristics, but longitudinal studies are required to determine whether patients transition between profiles or show stable or changing resilience trajectories over time.

### 4.2 Differences in psychosocial characteristics across latent classes of psychological resilience

The findings of this study indicate that patients in different latent classes of psychological resilience exhibit significant differences in anxiety, depression, quality of life, and social support, with a clear gradient pattern. Specifically, as psychological resilience increases, levels of anxiety and depression decrease, whereas global health status and family support increase. These results highlight the close association between psychological resilience and both negative emotional states and positive psychosocial resources. Consistent with previous research, cancer patients with higher levels of psychological resilience tend to report lower anxiety and depression and better quality of life. This may be explained by the role of psychological resilience in facilitating positive cognitive appraisal and effective coping strategies, thereby buffering the psychological impact of illness (25). In addition, psychological resilience has been shown to indirectly improve quality of life by influencing the pathways linking perceived stress and emotional responses (26). It is noteworthy that, in this study, family support differed significantly across latent classes, whereas support from friends and other sources did not. This finding suggests that, within the Chinese cultural context, family support may play a particularly important role in psychological adaptation. Notably, the absence of a statistically significant difference in the three-category marital-status variable should not be interpreted as evidence that relationship context is unrelated to resilience, because marital status was coarsely categorized and does not directly reflect the presence, quality, or availability of partner and family support. Previous studies have indicated that in family-oriented social structures, emotional support and caregiving involvement from family members are critical for promoting patients’ psychological recovery (27). Overall, psychological resilience in patients with brain glioma reflects not only individual psychological characteristics but also a key integrative construct linking emotional status, quality of life, and social support.

In this study, anxiety and depression were included in the regression model because the main analytical outcome was latent class membership derived from RS-14 items. Therefore, HADS-A and HADS-D scores were treated as external psychosocial correlates used to characterize the emotional profiles associated with different resilience classes. This approach should not be interpreted as assuming that anxiety and depression are unidirectional causes of resilience. The relationship between emotional distress and resilience may be bidirectional. Lower resilience may reduce patients’ capacity to cope with diagnostic uncertainty, neurological symptoms, and treatment burden, thereby increasing vulnerability to anxiety and depression. Conversely, persistent anxiety and depressive symptoms may weaken perceived coping ability, reduce motivation for active adjustment, and contribute to lower self-rated resilience. Therefore, the observed associations should be interpreted cautiously, and longitudinal studies are needed to clarify temporal and causal relationships among resilience, anxiety, and depression.

The present study builds upon a growing body of research on resilience in patients with brain tumors, further supporting its clinical relevance. Dinapoli et al. reported that, among patients with glioblastoma undergoing radiochemotherapy, higher spiritual well-being was associated with higher resilience and absence of psychosocial distress, and patients with greater resilience showed longer survival than those with lower resilience (28). Although the present study did not assess spiritual well-being or survival outcomes, our finding that higher resilience classes were characterized by lower anxiety and depression and better global health status is consistent with the view that resilience is closely related to emotional adaptation during glioma treatment. In addition, Maneekrong et al. found that symptom severity, social support, and treatment plan explained a substantial proportion of resilience among patients with brain tumors awaiting surgery, and emphasized the need to assess both physical and mental tumor-related symptoms and include caregivers in preoperative care (29). Similarly, the present study found that family support and disease-related factors were associated with resilience class membership, suggesting that resilience assessment in glioma patients should be integrated with symptom evaluation, caregiver involvement, and psychosocial support planning across different treatment stages.

### 4.3 Differences in psychological resilience in relation to demographic and disease characteristics

The present study demonstrated significant differences in age, educational attainment, household income, tumor grade, and recurrence status across latent classes of psychological resilience. Specifically, patients in the low psychological resilience group were more likely to be older, have lower income, and present with WHO grade III–IV glioma, whereas those in the high psychological resilience group were more likely to have higher educational attainment and higher income. However, the household-income categories used in this study represent relative socioeconomic strata within the study sample and should not be interpreted as formal definitions of poverty or affluence. These findings are consistent with previous studies in cancer populations, which have shown that individuals with higher educational attainment and better economic conditions tend to exhibit higher levels of psychological resilience, whereas disease severity is negatively associated with resilience (30). Taken together, these findings suggest that psychological resilience is shaped by the combined influence of available resources and disease-related stress. Educational attainment and income may reflect the cognitive and material resources accessible to individuals, whereas WHO grade III–IV status and recurrence may represent ongoing disease-related stressors. Under conditions of limited resources and sustained stress, individuals may be more likely to exhibit lower levels of psychological resilience, whereas those with greater resources may be better able to maintain stable psychological adaptation. These results further suggest that, in clinical nursing practice, psychological interventions alone may not be sufficient to improve adaptive outcomes in certain patient groups. For patients with limited resources, it may be necessary to address factors such as access to information, family support, and financial burden in parallel, as these factors may influence the effectiveness of psychological interventions (31).

The associations of family support, education, and income with resilience may be explained by differences in psychosocial and healthcare resources. Family support may provide emotional reassurance, daily caregiving, assistance with treatment decision-making, and continuity of support during hospitalization and subsequent treatment. Higher educational level may improve patients’ health literacy, understanding of disease-related information, communication with healthcare professionals, and ability to adopt active coping strategies. Higher household income may reduce treatment-related financial stress and improve access to rehabilitation, psychological counseling, transportation, and continuing care. Therefore, resilience differences observed in this sample may be related not only to individual psychological characteristics but also to differences in available supportive resources. In similar clinical settings, these findings may encourage clinicians to consider psychological assessment together with family support, social needs, and potential referral needs when evaluating patients with different resilience profiles.

### 4.4 Potential clinical and service implications

The present findings should primarily be interpreted as exploratory evidence from a single-center clinical sample rather than as population-level estimates. Within similar neuro-oncology settings, recognition of heterogeneity in psychological resilience may provide a useful clinical heuristic for identifying patients who could benefit from more detailed psychosocial assessment. In particular, patients characterized by relatively lower resilience together with greater emotional distress, limited family support, or greater disease burden may warrant closer clinical attention. However, the present findings do not establish a validated triage system and should not be used directly to determine service eligibility or resource allocation.

From a service perspective, the observed associations may help generate hypotheses regarding how resilience assessment could eventually be incorporated into supportive care pathways. Nevertheless, because participants were recruited by convenience sampling from a single tertiary hospital and were limited to clinically stable patients able to complete self-report questionnaires, the sample cannot be assumed to represent the broader glioma population. Accordingly, implications for psycho-oncology service design, population-level screening, or resource allocation require confirmation in larger, multicenter studies using more representative sampling strategies. Future longitudinal and implementation studies are also needed to determine whether resilience-informed screening or referral approaches improve access to psychosocial care or patient outcomes.

### 4.5 Limitations

This study has several limitations. First, it was a single-center cross-sectional study using convenience sampling among clinically stable inpatients who were able to complete self-report questionnaires. This sampling approach may have introduced selection bias because patients who were available, clinically stable, and capable of completing the questionnaires may differ systematically from the broader population of patients with glioma. Patients with severe neurological or cognitive impairment, critical illness, or communication difficulties may therefore have been underrepresented. Consequently, the observed class proportions and associations should not be regarded as population estimates, and the external validity and generalizability of the findings beyond similar clinical settings are limited. In addition, cognitive eligibility was based on functional clinical screening rather than a standardized cognitive instrument or formal neuropsychological assessment. Therefore, mild or subtle cognitive deficits that did not prevent questionnaire completion may not have been identified. Because cognitive status may itself be associated with psychological adaptation and self-reported resilience, residual confounding related to unmeasured cognitive function cannot be excluded. Furthermore, psychological resilience, anxiety, depression, quality of life, and perceived social support were assessed at a single time point. Consequently, temporal or causal relationships among these variables cannot be established, and potential changes in resilience across diagnosis, treatment, recurrence, rehabilitation, and follow-up were not captured. Accordingly, the latent classes identified in this study represent cross-sectional item-response profiles rather than longitudinal resilience trajectories, and no conclusions can be drawn regarding the stability, fluctuation, or transition of class membership over time. Although psychological or psychiatric support and the use of glucocorticoids, antiepileptic drugs, antidepressants, and anxiolytics were reviewed, only yes/no exposure during the index hospitalization was consistently available. Incomplete information regarding indication, dosage, duration, timing, adherence, and follow-up changes prevented a detailed evaluation of their potential influence on psychological resilience.

Marital status was also captured using a relatively coarse three-category classification that did not separately identify cohabiting or other non-marital partnerships and combined divorced and widowed participants. Because these relationship contexts may differ in both emotional consequences and the availability of interpersonal support, the nonsignificant association between marital status and resilience class membership should be interpreted cautiously. Future studies should collect more detailed information on partnership status, bereavement, and the actual availability and quality of partner support.

The study also relied primarily on self-report measures of conceptually related psychosocial constructs. Although multicollinearity diagnostics did not indicate serious multicollinearity, the observed associations should not be interpreted as independent causal effects. Moreover, the included instruments did not assess other potentially relevant factors, such as personality traits, coping strategies, illness perception, perceived control, spiritual well-being, caregiver burden, or brain tumor-specific neurological and functional concerns that could be evaluated using instruments such as the EORTC QLQ-BN20. Finally, latent class solutions are partly sample-dependent, and the mechanisms linking family support, education, income, disease severity, and psychological resilience were not directly examined. Larger multicenter longitudinal studies incorporating broader psychosocial and brain tumor-specific assessments are needed to validate the three-class structure, examine changes over time, clarify the underlying mechanisms, and determine whether resilience subtype-based screening or interventions can improve patient outcomes.

## 5 Conclusion

Based on latent class analysis, this study identified three latent classes of psychological resilience within a single-center clinical sample of patients with brain glioma, with differences across classes in anxiety, depression, quality of life, social support, and several demographic and disease-related characteristics. These classes should be interpreted as cross-sectional resilience profiles based on item-response patterns at a single assessment time point rather than as evidence of stable, fluctuating, or changing resilience trajectories over time. These findings provide exploratory evidence of heterogeneity in psychological resilience and may help clinicians in similar settings recognize patients who warrant more detailed psychosocial assessment and supportive attention. However, because the study used convenience sampling at a single tertiary hospital and included clinically stable patients capable of completing self-report questionnaires, the observed class structure, proportions, and associated factors should not be generalized directly to the broader glioma population. The findings should therefore be regarded as hypothesis-generating rather than as evidence for population-level screening, resource allocation, or psycho-oncology service design. Larger multicenter studies using more representative sampling strategies, together with prospective longitudinal and interventional studies, are needed to validate these findings and determine their clinical utility.

## Data Availability

The original contributions presented in the study are included in the article/supplementary material, further inquiries can be directed to the corresponding author.
